# MiR-146a is over-expressed and controls IL-6 production in cystic fibrosis macrophages

**DOI:** 10.1038/s41598-019-52770-w

**Published:** 2019-11-07

**Authors:** Francesco R. Luly, Manuella Lévêque, Valerio Licursi, Giuseppe Cimino, Corinne Martin-Chouly, Nathalie Théret, Rodolfo Negri, Luca Cavinato, Fiorentina Ascenzioni, Paola Del Porto

**Affiliations:** 1 Department of Biology and Biotechnology, “C. Darwin” Sapienza University, Rome, Italy; 2 Cystic Fibrosis Centre, Policlinico Umberto I, Rome, Italy; 3 Inserm, EHESP, Irset (Institut de recherche en santé, environnement et travail) – UMR S 1085, F-35000 University of Rennes, Rennes, France; 4 Institute of Molecular Biology and Pathology, Italian National Research Council, Rome, Italy; 5 Present Address: Service de Dermatologie CHU de Poitiers, University of Poitiers, Poitiers, France

**Keywords:** RNA sequencing, Inflammation

## Abstract

Cystic fibrosis (CF) is an inherited disease that is characterised by susceptibility to bacterial infections and chronic lung inflammation. Recently, it was suggested that macrophages contribute to impaired host defence and excessive inflammatory responses in CF. Indeed, dysfunction attributed to CF macrophages includes decreased bacterial killing and exaggerated inflammatory responses. However, the mechanisms behind such defects have only been partially defined. MicroRNAs (miRNAs) have emerged as key regulators of several macrophage functions, including their activation, differentiation and polarisation. The goal of this study was to investigate whether miRNA dysregulation underlies the functional abnormalities of CF macrophages. MiRNA profiling of macrophages was performed, with 22 miRNAs identified as differentially expressed between CF and non-CF individuals. Among these, miR-146a was associated with significant enrichment of validated target genes involved in responses to microorganisms and inflammation. As miR-146a dysregulation has been reported in several human inflammatory diseases, we analysed the impact of increased miR-146a expression on inflammatory responses of CF macrophages. These data show that inhibition of miR-146a in lipopolysaccharide-stimulated CF macrophages results in increased interleukin-6 production, which suggests that miR-146a overexpression in CF is functional, to restrict inflammatory responses.

## Introduction

Cystic fibrosis (CF) is an autosomal genetic disease that is caused by mutations in the CF transmembrane conductance regulator (*CFTR*) gene^1^. CFTR is an epithelial channel that regulates anion transport and mucociliary clearance in the airways, and its absence results in mucus plugging and chronic bacterial infections, the persistence of which can lead to chronic inflammation and tissue destruction, and finally to respiratory failure^2,3^. In recent years, it has emerged that the lack of control of both infections and excessive inflammatory responses in CF patients is due, at least in part, to dysfunction of the innate immune system^4^. In particular, abnormalities in several functions of human macrophages in CF have been reported, including altered phagocytosis and decreased killing of lung pathogens, such as *Pseudomonas aeruginosa* and *Burkholderia cepacia*^5–9^. Moreover, CFTR deficiency in human macrophages has been associated with exaggerated inflammatory responses to bacterial stimuli and defective M2 polarisation^10,11^.

MicroRNAs (miRNAs) are short non-coding RNA molecules that usually comprise 18 to 25 nucleotides that can inhibit expression of target genes by binding to complementary sequences in the 3′-untranslated regions of mRNAs. This targets these molecules to translational repression or degradation^12^. MiRNAs have emerged as key regulators of several aspects of macrophage biology, including their activation, differentiation and polarisation. Accordingly, M1 and M2 macrophages appear to be dominated by different miRNAs. MiR-155 is highly expressed in classical activated macrophages (M1), where it contributes to drive M1 polarisation by directly targeting interleukin (IL)-13 receptor α1, and it indirectly inhibits expression of M2-related genes^13^. In contrast, miR-511-3p and mir-223 have been shown to promote M2 macrophage polarisation. Mir-511-3p targets Rho-associated coiled-coil containing protein kinase 2 (Rock2), which is a serine threonine kinase that phosphorylates IRF4, and thus promotes the expression of M2-related genes^14^. MiR-223 targets the transcription factor Pknox1, which suppresses the pro-inflammatory activation of macrophages^15^.

In addition, following macrophage activation, miRNAs contribute to prompt induction of inflammation and its rapid resolution through a complex network of interactions^16^. Accordingly, in a murine model of acute inflammation, it was shown that an NF-κB–miRNAs network controls the robustness of the inflammatory response and its time-dependent restriction. In particular, it has been proposed that the temporal asymmetry of miR-146a and miR-155 production and activity creates the correct balance between positive and negative regulators of NF-κB, which in turn regulates inflammatory responses. Indeed, it has been reported that miR-155 is rapidly and highly transcribed in response to an inflammatory stimulus, and that through repression of SHIP1 and SOCS1, miR-155 amplifies NF-κB activity. As an inflammatory response develops, miR-146a levels accumulate, which leads to repression of IRAK1 and TRAF6, and to subsequent attenuation of NF-κB activation. Thus, it appears that not only the miRNA levels, but also their temporal induction are important in the correct regulation of inflammatory responses.

To determine whether miRNA dysregulation indeed underlies the functional abnormalities of CF macrophages, we performed miRNA profiling in macrophages from CF and non-CF individuals. This led to the identification of a panel of differentially expressed miRNAs in CF macrophages, as compared to non-CF macrophages. Among these, miR-146a was associated with significant enrichment of validated target genes that are involved in responses to microorganisms and inflammation. In addition, as miR-146a dysregulation has been reported for several human inflammatory diseases, we analysed the impact of increased miR-146a expression on the inflammatory response of CF macrophages^17–19^. These data show that inhibition of miR-146a in lipopolysaccharide (LPS)-stimulated CF macrophages results in increased IL-6 production, which suggests that the function of miR-146a overexpression in CF is to restrict the inflammatory response.

## Results

### MiRNA profiling in CF macrophages and in-silico functional analysis

**Figure 1:**
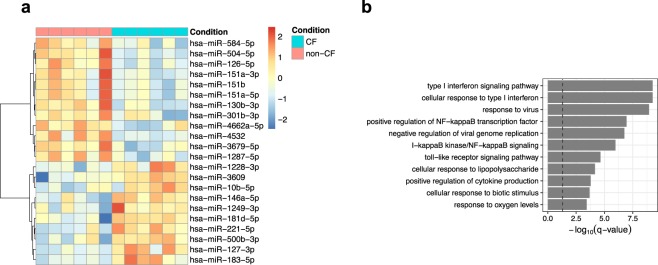
MiRNA profiling in CF and non-CF macrophages. (**a**) Heatmap showing the differentially expressed miRNAs. Each row indicates the expression level of the indicated single miRNA (z-score scaled), and each column is from one single subject. The row dendrogram represents the miRNA clustering using the hierarchical clustering method, with the Pearson correlations as the similarity measure. (**b**) Enriched categories of the miR-146a target genes. Bar plot showing the top significantly enriched categories of the GO biological processes of the miR-146a target genes. X-axis, significance of the categories; bars are ordered according to their −log_10_(q-value); dashed line, −log_10_(0.05) value.

MiRNA-specific RNA sequencing was performed in monocyte-derived macrophages from six patients with CF and from six age- and sex-matched non-CF individuals (controls). In this analysis, 785 miRNAs were identified and quantified in these macrophages from CF patients and controls. The most differentially expressed miRNAs were selected according to the following criteria: threshold of ≥1.5-fold or ≤−1.5-fold change, and false-discovery-rate-adjusted *P* (*q*) ≤ 0.05. From this analysis, 22 miRNAs that were differentially expressed in CF vs non-CF macrophages (10 up-regulated, 12 down-regulated) were selected (Fig. 1a).

To predict potential targets and to allow functional analysis of the differentially expressed miRNAs, *in-silico* analysis was performed, which included miRNA–target interactions, Gene Ontology (GO) enrichment, and network interactions. Thus, each of the 22 selected miRNAs was subjected to analysis of their miRNA targets, using a manually curated database of experimentally validated microRNA–target interactions: miRTarBase^20^. Next, for the validated targets, enrichment analysis of the GO terms in biological processes and the Reactome pathways was performed^21,22^.

Through this approach, a significant number of validated targets was identified from miRTarBase for a subset of the 22 differentially expressed miRNAs, and significantly enriched categories (FDR < 0.1) were identified for five of the up-regulated miRNAs and six of the down-regulated miRNAs (Supplementary Figs S1 and S2). Collectively, each miRNA defined a unique set of categories that comprised many different terms and pathways. Among the differentially expressed miRNAs, miR-146a was the only one that showed enrichment in GO terms related to cellular responses to pathogens (Fig. 1b; Supplementary Fig. S1A). In accordance, the Reactome analysis confirmed the involvement of miR-146a in the Toll-like receptor (TLR) cascade, and in interferon and interleukin signalling (Supplementary Fig. S1B).

### MiR-146a is up-regulated in CF macrophages

Among the differentially expressed miRNAs in the CF macrophages, miR-146a was the miRNA mostly implicated in the regulation of inflammation in innate immune cells, as determined by the GO terms and the Reactome enrichment analyses. Thus, considering the hyper-inflammatory phenotype of the CF macrophages, miR-146a was selected for further investigation^23,24^.

**Figure 2:**
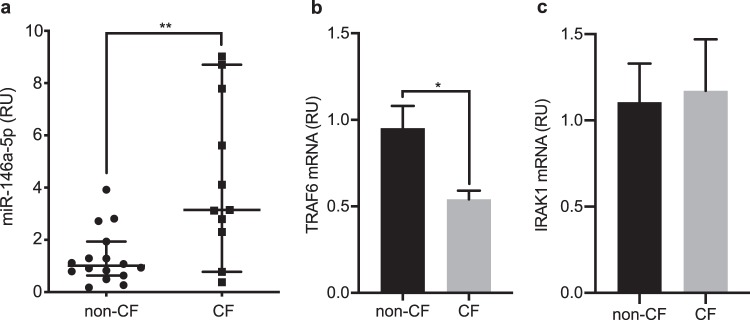
Up-regulation of miR-146a and down-regulation of TRAF6 mRNA in CF macrophages. (**a**) MiR-146a levels were measured using RT-qPCR in non-CF (n = 16) and CF (n = 11) macrophages. The data were normalised to expression of the RNU6B endogenous control. Each symbol represents a single individual. **p < 0.01 (Mann-Whitney tests) (**b**,**c**) TRAF6 and IRAK1 mRNAs were evaluated using RT-qPCR in non-CF (n = 8) and CF (n = 8) macrophages. The data were normalised to expression of the endogenous β-actin control. Data are means ± SEM. *p < 0.05 (unpaired t-tests).

To validate the sequencing data, the analysis of miR-146a expression was extended to macrophages from 11 CF and 16 non-CF individuals, using RT-qPCR. These data are reported in Fig. 2a, and they show median miR-146a expression for CF macrophages as 3.11-fold that for non-CF macrophages (*p* = 0.0056), which confirms its significant up-regulation in these CF macrophages.

As negative regulators with short mRNA-binding sequences, miRNAs have the potential to regulate numerous target genes. To investigate whether miR-146a up-regulation had any effects on the expression of its targets in CF macrophages, the expression was examined for two of the most relevant validated miR-146a targets, *IRAK1* and *TRAF6*, which are crucial adaptors for TLR-mediated NF-κB signalling^25^. Determination of TRAF6 and IRAK1 mRNA levels in these CF and non-CF macrophages using RT-qPCR revealed that the CF macrophages had significantly lower levels of TRAF6 mRNA (*p* = 0.0103) than the non-CF macrophages, but similar levels of IRAK1 mRNA (Fig. 2b,c).

Thus, these data demonstrated that increased miR-146a in CF macrophages is associated with mRNA decrease of at least one of its validated targets, TRAF6.

### MiR-146a inhibition in macrophages

**Figure 3:**
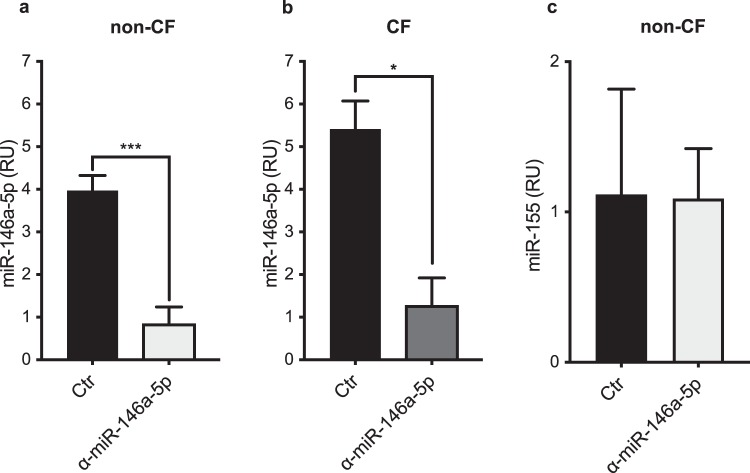
Knock-down of miR-146a in non-CF and CF macrophages. (**a**,**b)** MiR-146a expression level in LPS-stimulated non-CF and CF macrophages. **(c)** MiR-155 expression in LPS-stimulated non-CF macrophages. MiRNAs were quantified 16 h after transfection with either the miR-146a inhibitor (α-miR-146a-5p) or the control inhibitor (Ctr) (n = 3). The data were normalised to expression of the endogenous RNU6B control. Data are means ± SEM. *p < 0.05; ***p < 0.001 (unpaired t-tests).

**Figure 4:**
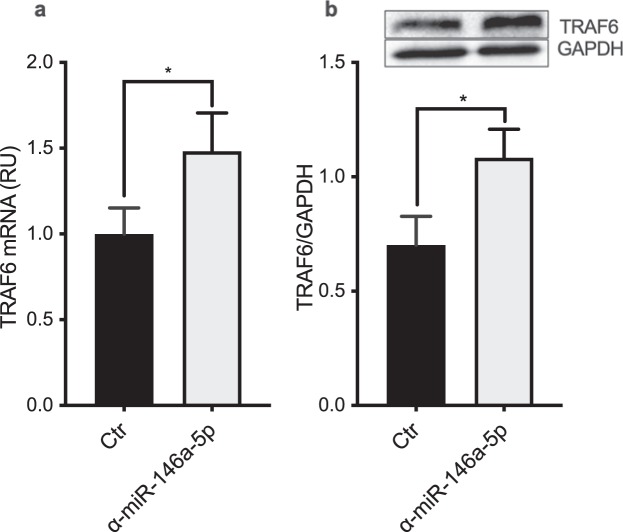
MiR-146a inhibition increases TRAF6 mRNA and protein levels in non-CF macrophages. (**a)** TRAF6 mRNA was evaluated using RT-qPCR in LPS-stimulated non-CF macrophages 8 h after transfection with the miR-146a inhibitor (α-miR-146a-5p) or the control inhibitor (Ctr) (n = 6). **(b)** TRAF6 protein expression 16 h after transfection. TRAF6 was assessed by Western blotting, with GAPDH as the endogenous control. Densitometric quantification and one representative out of three independently performed experiments (inset) are shown. Data are means ± SEM; *p < 0.05 (paired t-tests).

MiR-146a is known to promote resolution of inflammation in response to LPS^16^. To functionally assess the role of miR-146a in these CF macrophages, the response to LPS was examined for the macrophages following inhibition of endogenous miR-146a. To accomplish this, the macrophages were stimulated for 2 h with 100 ng/mL LPS, and then transfected with either miR-146a or a control inhibitor at 50 nM, as previously reported^16^. The knock-down efficiency was determined through evaluation of miR-146a expression in the non-CF and CF macrophages using RT-qPCR. As shown in Fig. 3, miR-146a was down-regulated to a similar degree in both the non-CF and CF macrophages. In particular, 16 h after transfection and compared to the control inhibitor, the miR-146a reduction was about 78% and 76% in these non-CF and CF macrophages, respectively (non-CF, *p* = 0.0007; CF, *p* = 0.0477; Fig. 3a,b). The expression of the unrelated miR-155 was not affected by the miR-146a inhibitor, which confirmed the specificity of this miR-146a inhibition (Fig. 3c). In addition, RT-qPCR and Western blotting of non-CF macrophages transfected with the miR-146a inhibitor demonstrated that miR-146a inhibition was associated with significant increases in *TRAF6* mRNA (*p* = 0.0395) and protein expression (*p* = 0.0273), compared to the control cells (Fig. 4a,b, Supplementary Fig. 3). At variance with this, analysis of IRAK1 protein expression did not show any significant differences between non-CF macrophages transfected with miR-146a or the control inhibitor (data not shown).

### MiR-146a reduces IL-6 production in CF macrophages

Based on previous data in murine models that have shown that miR-146a modulates IL-6 and IL-10 production in response to LPS, the effects of miR-146a inhibition on the production of IL-6, IL-10 and IL-8 were compared in CF macrophages transfected with the miR-146a inhibitor^16,26^. Here, macrophages isolated from seven CF patients and seven non-CF controls were stimulated with LPS and transfected with the miR-146a inhibitor, and the production of these cytokines was quantified in the supernatants using ELISA.

**Figure 5:**
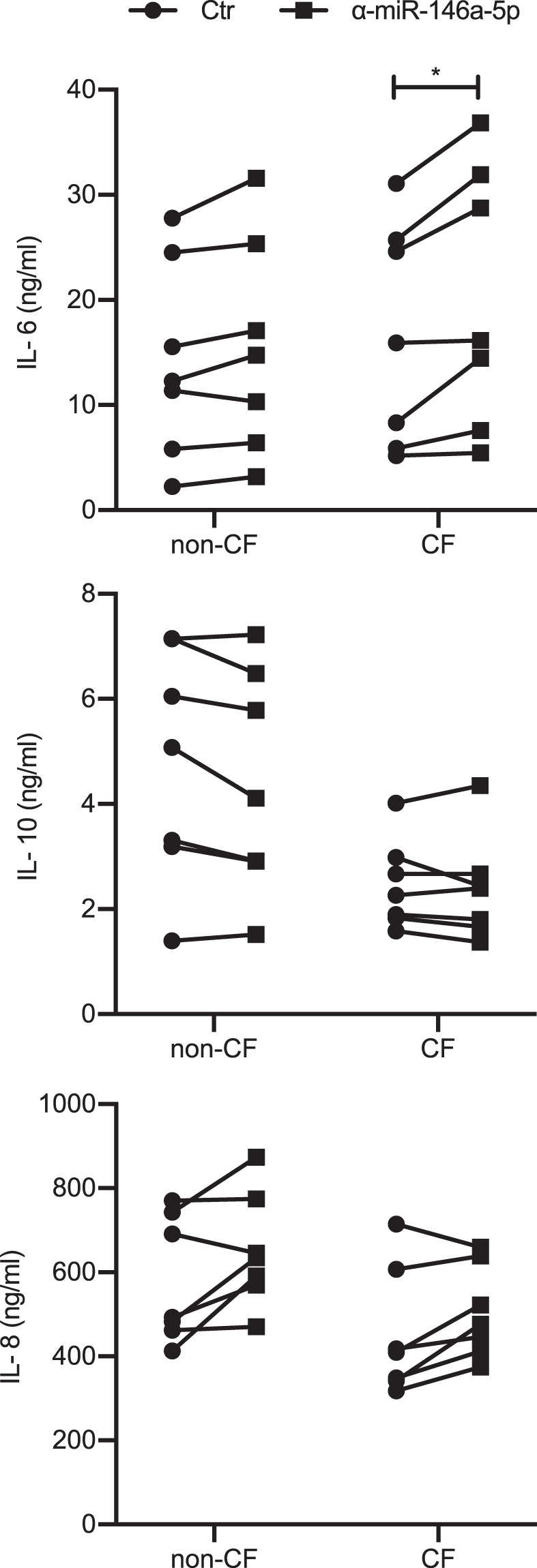
Increased production of IL-6 by miR-146a knock-down in CF macrophages in response to LPS. Cytokines produced by LPS-stimulated macrophages transfected with the miR-146a inhibitor (α-miR-146a-5p) or the control inhibitor (Ctr). IL-6 (top), IL-10 (middle) and IL-8 (bottom) were quantified in the supernatants of non-CF (n = 7) and CF (n = 7) macrophages 24 h after transfection, using ELISA. *p < 0.05 (Wilcoxon tests).

**Figure 6:**
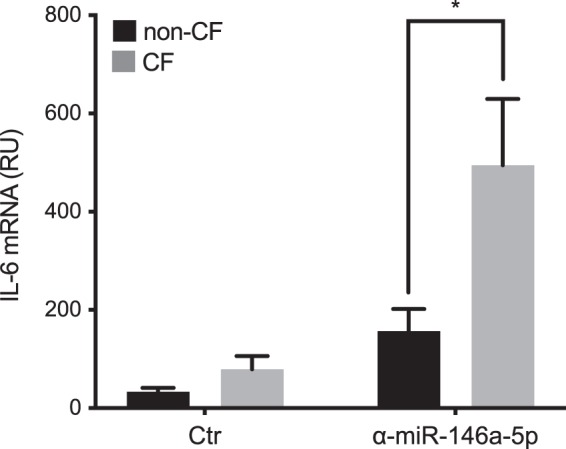
Increased IL-6 mRNA levels in miR-146a knock-down CF macrophages in response to LPS. Relative quantification of IL-6 mRNA in LPS-stimulated non-CF (n = 5) or CF (n = 5) macrophages transfected with the miR-146a-5p inhibitor (α-miR-146a-5p) or the control inhibitor (Ctr). Data are means ± SEM; *p < 0.05 (unpaired t-tests).

These data are reported in Fig. 5, and they demonstrated that inhibition of miR-146a in CF macrophages led to significant increases in production of IL-6, with respect to the same CF macrophages treated with the control inhibitor (*p* = 0.0156); however, this did not significantly affect the cytokine production in non-CF macrophages. In contrast, inhibition of miR-146a did not have any effects on the production of IL-10 and IL-8 by either the CF or the non-CF macrophages, which suggests that the anti-inflammatory effects of miR-146a on IL-6 production are specific (Fig. 5). Further support for the role of miR-146a in the modulation of IL-6 production in LPS-stimulated CF macrophages was shown by the evidence that the inhibition of miR-146a leads to significant increases in IL-6 mRNA levels in the CF macrophages, as compared to the non-CF macrophages (Fig. 6, *p* = 0.045).

Taken together, these data show that in CF macrophages, miR-146a controls IL-6 production in response to bacterial stimuli.

## Discussion

At present, there is general consensus on the involvement of immune cells in the progression of cystic fibrosis, and in particular of phagocytic cells^27^. This relatively new concept is supported by the demonstration that human monocytes and macrophages express CFTR and that phagocytosis and inflammatory responses to bacteria are altered in CF macrophages^28–30^. It is therefore possible that macrophage dysfunction contributes to disease progression and severity by impairing the ability of CF patients to clear lung infections and by sustaining inflammation. Although many studies have reported on the functional abnormalities of CF macrophages, the molecular mechanisms that underlie such defects have not been fully elucidated to date.

Evidence that has accumulated over the last decade has suggested that miRNAs are key regulators of multiple processes of macrophage biology, which includes their differentiation, infiltration and activation^31^. Thus, to determine whether dysregulation of miRNA expression occurs in CF macrophages, we performed global miRNA profiling of macrophages from non-CF and CF individuals, and used computational analysis to identify 22 differentially expressed miRNAs in the CF macrophages compared to the control macrophages. Similarly, in well-differentiated primary cultures of human CF airway epithelia cells and in CF bronchial brushing, altered expression of numerous microRNAs has been reported^32,33^. In these previous studies, miR-145, miR-223, miR-494 and miR-509-3p were shown to be over-expressed in CF cells compared to controls, and their expression was inversely correlated with CFTR levels, which suggested that these miRNAs can directly regulate CFTR expression. Furthermore, aberrant expression of miRNAs involved in the regulation of inflammatory pathways has been observed in CF epithelial cells. In CF bronchial brushings, down-regulation of miR-126 was correlated with significant up-regulation of Target of Myb1 (TOM1), a Tollip-binding protein that negatively regulates TLR2, TLR4 and IL-1RI signalling^34^. More recently, a parallel miRNome and transcriptome analyses of primary *in-vitro* differentiated epithelial cells from CF and non-CF individuals demonstrated that about half of the dysregulated miRNAs in CF samples was predicted to be associated with the NF-κB pathway. In particular, down-regulation of miR-199a-3p inversely correlated with increased expression of IKKβ and IL-8 in CF cells^35^. In the present study, our *in-silico* approaches identified common biological processes and molecular pathways that are associated with dysregulated miRNAs in CF macrophages, whereby we identified miR-146a as the miRNA mainly involved in inflammation.

MiR-146a dysregulation in innate immune cells has been implicated in several human inflammatory diseases, including rheumatoid arthritis, systemic lupus erythematosus and Sjogren’s syndrome, which has suggested a role for miR-146a in CF macrophages^17–19^. Here, we have shown that expression of miR-146a was consistently and reproducibly increased in CF macrophages *versus* non-CF, and that it was associated with lower levels of its validated target TRAF6. Indeed, among the several miR-146a target genes in immune cells, which include *CXCR4*, *STAT1*, *IRAK1* and *TRAF6*, the last two of these have been the most frequently validated^36,37^. Dysregulation of miR-146a has been previously documented in CF. Indeed, Montanini *et al*. demonstrated that miR-146a was up-regulated in CF serum, and that its dysregulation was related to the onset of CF-related diabetes^38^. Moreover, miRNA profiling in peripheral blood mononuclear cells cultured with CF plasma identified miR-146a as one of the top-ranked up-regulated miRNAs^39^. A significant increase in miR-146a expression has also been reported in peripheral blood mononuclear cells and monocytes from rheumatoid arthritis patients, but no differences in the levels of TRAF6 and IRAK1 were shown between patients and controls, thus leading to the hypothesis that defective negative regulation of the target genes by miR-146a contributes to sustained pro-inflammatory cytokine production in patients^18^.

At variance with this, the evidence from the present study that inhibition of miR-146a in CF macrophages leads to significant increases in the production of IL-6 indicates that miR-146a is functional and contributes to the limiting of IL-6 production in these macrophages. It is interesting to note that miR-146a inhibition did not significantly affect the production of IL-6 in the control macrophages stimulated with LPS. These data are in agreement with previous findings that have shown that miR-146a inhibition does not significantly alter IL-6 production by the human monocytic THP1 cell line in response to LPS stimulation^19^.

There are several possibilities to explain why miR-146a specifically modulates IL-6 production in CF macrophages and does not appear to do the same in the cells from healthy donors. First, it might be a direct consequence of the increased expression of miR-146a in the CF macrophages, which through target-gene modulation can re-programme the cellular responses to endotoxins. Indeed, it has been shown that miR-146a^−/−^ bone-marrow-derived dendritic cells pre-treated with exosomes isolated from wild-type cells produce less IL-6 following LPS stimulation than cells that received exosomes from miR-146a^−/−^ mice^26^. This suggests that even the modest increase in miR-146a as that induced by exosome transfer can change the immune responses of recipient cells.

Alternatively, it can be speculated that in CF macrophages, miR-146a up-regulation is functional to down-regulate the otherwise hyper-production of LPS-induced IL-6 that is due to an increase in TLR4 signalling in the CF macrophages^23^. In CF macrophages, dysregulation of TLR signalling has been attributed to defective up-regulation of Caveolin-1, which allows trafficking of HO-1 to the caveolae in a p38 MAPK-dependent manner, and leads to down-regulation of TLR-4 pro-inflammatory signalling. Reduced Caveolin-1 expression was caused by high miR-199a-5p levels as a consequence of blunted phosphatidylinositol 3-kinase/ protein kinase B (AKT) pathway in CF macrophages^40^.

Together with the evidence that miR-146a is up-regulated and contributes to the limiting of IL-6 production in CF macrophages, these data underline the importance of miRNAs in shaping inflammatory responses in CF macrophages. In particular, in response to LPS stimulation, IL-6 production by CF macrophages appears to be the result of simultaneous and opposing actions of pro-inflammatory and anti-inflammatory miRNAs, where their dysregulated expression leads to reduced or enhanced TLR signalling through modulation of receptor-associated molecules.

## Methods

### Study subjects

Sixteen patients with CF (from the Regional Cystic Fibrosis Centre, Sapienza University, Rome Italy) that was confirmed by positive sweat tests and genotyping were enrolled in the study. Fourteen patients were F508del homozygous, and two were F508del/L558S and F508del/E409X. Blood samples from 18 age- and sex-matched healthy donors were used as controls.

MiRNA sequencing was performed for the macrophages from six CF individuals (three females, three males; five F508del/F508del, one F508del/L558S; mean age, 35.0 ± 14 years) and six sex-matched controls (mean age, 35.3 ± 11). All of the patients were clinically stable and blood samples were collected for isolation of CD14^+^ cells when they attended the clinic for routine evaluations. Informed written consent was obtained from all of the participants after approval of the study by the local Ethics Committee (Comitato Etico, Azienda Policlinico Umberto I, Rome, Italy; 1233/2016). All of the methods were carried out in accordance with the relevant guidelines and regulations.

### Isolation and differentiation of human monocytes

Macrophages were generated as previously described^41^. In brief, CD14^+^ cells were purified from peripheral blood mononuclear cells by positive selection with an anti-CD14 monoclonal antibody coupled to magnetic beads (Miltenyi Biotec, Bergisch Gladbach, Germany). CD14^+^ cells were differentiated for 7 days in RPMI 1640 (Gibco-BRL, Invitrogren Corporation Carlsbad, CA, USA) supplemented with 20% foetal calf serum and 100 ng/mL recombinant macrophage-colony stimulating factor (PeproTech Inch, Rocky Hill, NY, USA).

### RNA extraction and real-time PCR

The isolation of total RNA and the DNAse treatments were performed using miRNeasy kits and RNAse-free DNAse sets, respectively (Qiagen, Germany). For miRNA quantification, total RNA was reverse transcribed using miScript II RT kits (Qiagen, Germany), and real-time qPCR was performed with miScript SYBR Green PCR kits and miScript primer assays for miR-146a-5p (MS00003535; Qiagen, Germany) and miR-155 (MS00031486; Qiagen, Germany). For mRNAs, total RNAs were reverse transcribed with Reverse Transcription System kits (Promega, USA) and amplified using PowerUp SYBR Green Master Mix (Applied Biosystems, USA). The endogenous controls were RNU6B (MS00033740; Qiagen, Germany) and β-actin. The following primers were used: IRAK1-F (5′-TGAGGAACACGGTGTATG-3′), IRAK1-R (5′-GTTTGGGTGACGAAACCTGGA-3′), TRAF6-F (5′-TTGCCATGAAAAGATGCAGAGG-3′), TRAF6-R (5′-CGCTCAAACTATGAACAGCCTG-3′), IL-6-F (5′-AGGCACTGGCAGAAAACAAC-3′), IL-6-R (5′-TTTTCACCAGGCAAGTCTCC-3′), β-ACTIN-F (5′-GCCGGGACCTGACTGACTA-3′), β-ACTIN-R (5′-TGGTGATGACCTGGCCGT-3′).

### MicroRNA-Seq

MicroRNA-Seq library preparation and sequencing was performed by IGA Technology Services (Udine, Italy) using the Illumina TruSeq Small RNA Library Preparation kits (Illumina, San Diego, USA), according to manufacturer instructions. An average number of 15 million reads was obtained per sample. The reads quality was evaluated using FastQC (version 0.11.2; Babraham Institute, Cambridge, UK). After adapter cleaning, the resulting reads ranged from 94.7% to 97.7%. A very high percentage (98.4%-99.2%) of these reads was mapped to the human genome (hg38 build; Genome Reference Consortium Human GRCh38) using the *sRNAbench* command line tool with ‘genome mapping mode’ from the *sRNAtoolbox* suite of software^42^.

Briefly, the *sRNAbench* pipeline first aligns all of the reads to the reference genome using the *Bowtie* aligner, then the coordinates are compared to the miRBase annotation version 21^43,44^. A read is assigned to the reference RNA if its coordinates lie completely within the chromosome coordinates of the reference RNA. Subsequently, the differentially expressed microRNAs analysis was performed with the *sRNAde* pipeline from the *sRNAtoolbox* suite. The module generates an expression matrix and uses the R/Bioconductor package *edgeR* to infer differential expression^45^. By using *edgeR*, *sRNAbench* applies TMM normalisation for detection of differentially expressed microRNAs, which has been reported to be among the most stable of the methods available^46^. To understand the biological meaning of the differentially expressed microRNAs, we performed an enrichment analysis of experimentally validated microRNA–target interactions from the manually curated miRTarBase database^20^. Enrichment analysis was performed with hypergeometric tests from Bioconductor R package clusterProfiler using the GO Biological Process and the Reactome database categories^21,22,47^.

The miRNA-seq raw data files have been deposited in the NCBI Gene Expression Omnibus under GEO: GSE134702 (https://www.ncbi.nlm.nih.gov/geo/query/acc.cgi?acc=GSE134702).

### MiRNA inhibitor transfection

Macrophages were cultured in RPMI 1640 with 20% foetal calf serum and 100 ng/mL macrophage-colony stimulating factor. On day seven, 3 × 10^5^ cells were stimulated with LPS (100 ng/mL). After 2 h of stimulation, the cells were transfected with either miR-146a or the control inhibitor (Exiqon, Denmark), at a final concentration of 50 nM, using Lipofectamine RNAiMAX Transfection Reagent (13778; Invitrogen, USA), according to the manufacturer instructions. The supernatants were removed and stored at –80 °C for cytokine quantification, and the cells were collected for RNA extraction and quantitative PCR analysis.

### Immunoblotting

Proteins were isolated using lysis buffer containing 50 mM HEPES, 150 mM NaCl, 1% Triton X100, 20 mM EDTA, and added protease inhibitor cocktail. Total protein levels were quantified using Bio-Rad protein assays, and equal amounts of protein were loaded and separated using 10% SDS–PAGE, followed by immunoblotting with the appropriate antibodies. These antibodies included: anti-TRAF6 (D21G3; Cell Signaling Technology, USA) diluted 1:1000, and anti-GAPDH (sc-25778; Santa Cruz, USA) diluted 1:400. The membranes were then washed, incubated with horseradish-peroxidase-linked secondary antibodies (GE Healthcare, UK), and visualised (Chemi Doc XRS system; Bio-Rad Laboratories Ltd, Hemel Hempstead, UK). Western blotting was quantified using the ImageJ software.

### ELISA

The IL-6, IL-10 and IL-8 in cell supernatants were quantified using ELISA kits (R&D Systems, UK), following the manufacturer instructions. The sensitivity of these tests was 9.38 pg/mL, 31.3 pg/mL and 31.3 pg/mL, respectively.

### Statistics

All of the statistical analyses were carried out in the Graphpad Prism software using Student’s t-tests, Mann-Whitney tests and Wilcoxon tests. *p* ≤ 0.05 was considered significant. In the Figures, statistically significant differences are indicated for *p* ≤ 0.05 (*), *p* ≤ 0.01 (**) and *p* ≤ 0.001 (***).

## Supplementary information

Supplementary Info SREP-19-31623A

## Data Availability

The datasets generated and analysed during the current study are available from the corresponding author on reasonable request.
