# Supplementary material for: MiR-146a is over-expressed and controls IL-6 production in cystic fibrosis macrophages
Source: Sci Rep. 2019 Nov 7;9:16259. doi: 10.1038/s41598-019-52770-w (PMC6838115; doi:10.1038/s41598-019-52770-w)
Supplement: Supplementary file 1 — Supplementary Info SREP-19-31623A [file 41598_2019_52770_MOESM1_ESM.pdf]

Supplementary Information

Manuscript title: MiR-146a is over-expressed and controls IL-6 production in cystic fibrosis macrophages

Authors: Francesco R. Luly, Manuella Lévêque, Valerio Licursi, Giuseppe Cimino, Corinne Martin-Chouly, Nathalie Théret, Rodolfo Negri[1], Luca Cavinato[1], Fiorentina Ascenzioni[1], Paola Del Porto[1]*.

Supplementary Figure S1

A

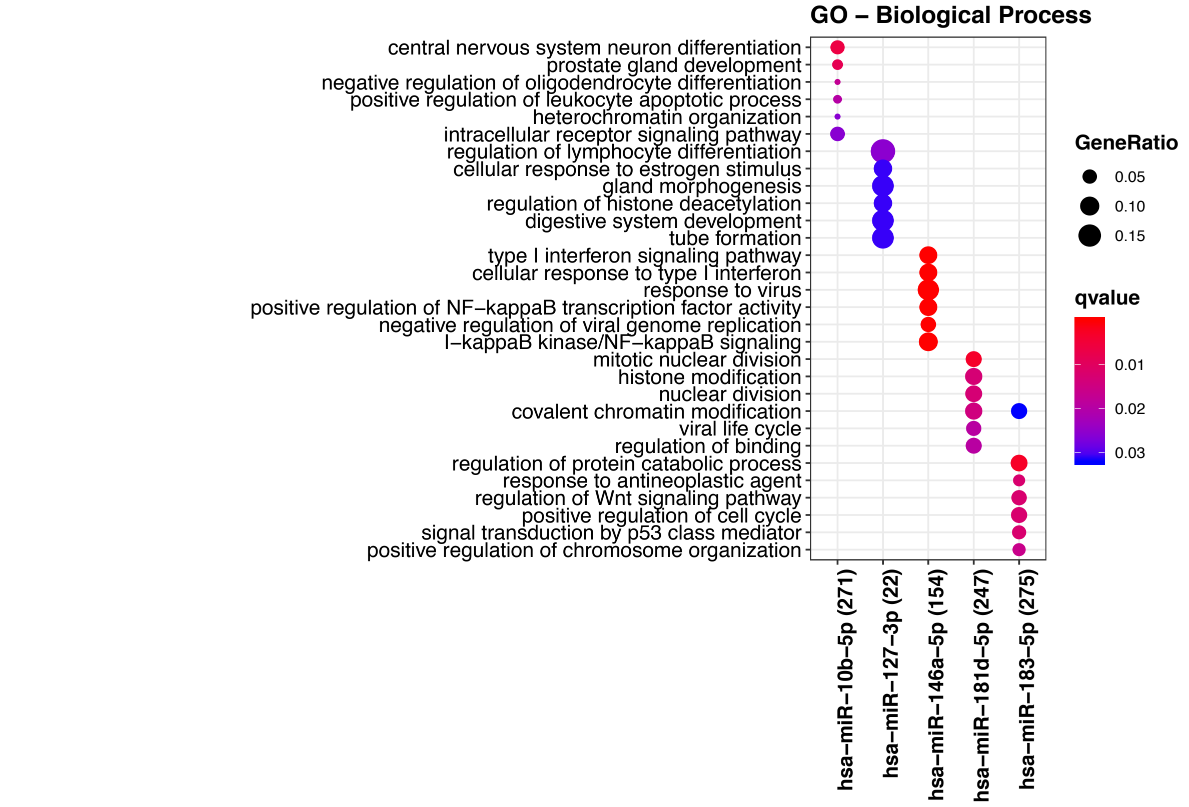

GO – Biological Process
central nervous system neuron differentiation
prostate gland development
negative regulation of oligodendrocyte differentiation
positive regulation of leukocyte apoptotic process
heterochromatin organization
intracellular receptor signaling pathway
regulation of lymphocyte differentiation
cellular response to estrogen stimulus
gland morphogenesis
regulation of histone deacetylation
digestive system development
tube formation
type I interferon signaling pathway
cellular response to type I interferon
response to virus
positive regulation of NF–kappaB transcription factor activity
negative regulation of viral genome replication
I–kappaB kinase/NF–kappaB signaling
mitotic nuclear division
histone modification
nuclear division
covalent chromatin modification
viral life cycle
regulation of binding
regulation of protein catabolic process
response to antineoplastic agent
regulation of Wnt signaling pathway
positive regulation of cell cycle
signal transduction by p53 class mediator
positive regulation of chromosome organization
hsa–miR–10b–5p (271)
hsa–miR–127–3p (22)
hsa–miR–146a–5p (154)
hsa–miR–181d–5p (247)
hsa–miR–183–5p (275)
GeneRatio
0.05
0.10
0.15
qvalue
0.01
0.02
0.03

B

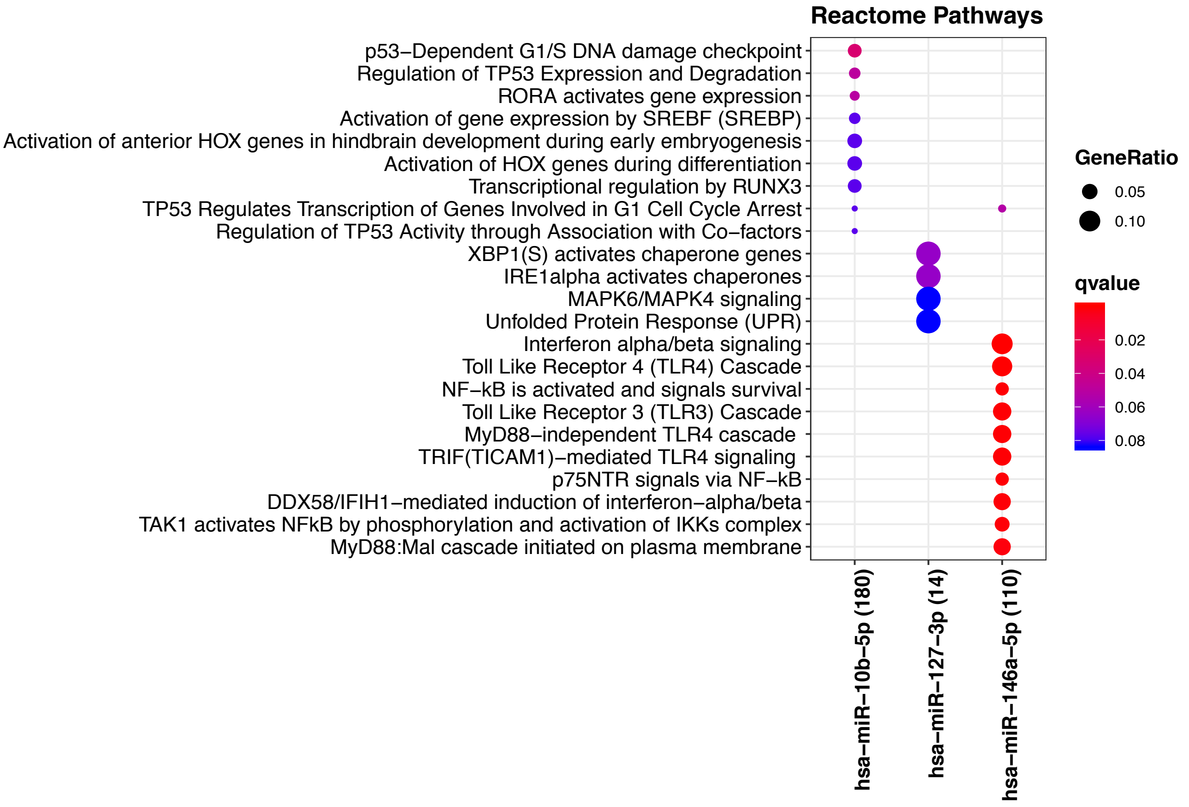

Reactome Pathways
p53–Dependent G1/S DNA damage checkpoint
Regulation of TP53 Expression and Degradation
RORA activates gene expression
Activation of gene expression by SREBF (SREBP)
Activation of anterior HOX genes in hindbrain development during early embryogenesis
Activation of HOX genes during differentiation
Transcriptional regulation by RUNX3
TP53 Regulates Transcription of Genes Involved in G1 Cell Cycle Arrest
Regulation of TP53 Activity through Association with Co–factors
XBP1(S) activates chaperone genes
IRE1alpha activates chaperones
MAPK6/MAPK4 signaling
Unfolded Protein Response (UPR)
Interferon alpha/beta signaling
Toll Like Receptor 4 (TLR4) Cascade
NF–kB is activated and signals survival
Toll Like Receptor 3 (TLR3) Cascade
MyD88–independent TLR4 cascade
TRIF(TICAM1)–mediated TLR4 signaling
p75NTR signals via NF–kB
DDX58/IFIH1–mediated induction of interferon–alpha/beta
TAK1 activates NFkB by phosphorylation and activation of IKKs complex
MyD88:Mal cascade initiated on plasma membrane
hsa–miR–10b–5p (180)
hsa–miR–127–3p (14)
hsa–miR–146a–5p (110)
GeneRatio
0.05
0.10
qvalue
0.02
0.04
0.06
0.08

Figure S1. Functional enrichment analysis of genes targeted by upregulated miRNAs.

(**A-B**) The dotplot shows the top significantly enriched categories of GO biological processes of the differentially upregulated miRNAs target genes. The total number of target genes found in miRTarBase database for each miRNA is reported on x axis labels. Size dots is correlated with the ratio between the total number of target genes and the number of genes that belongs to a GO (**A**) or Reactome (**B**) category. Dots are colored according the Benjamini-Hochberg false discovery rate adjusted p-values (q value) from blue (higher q value) to red (lower q value).

Supplementary Figure S2

A

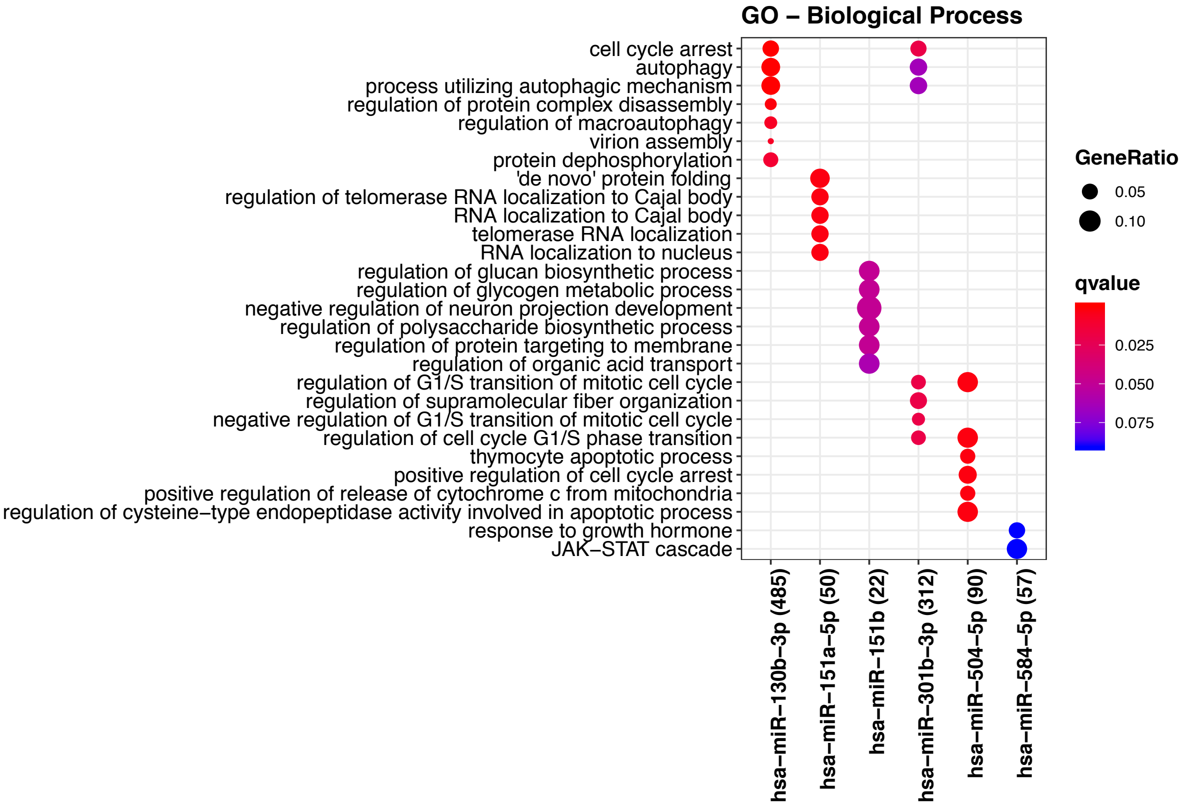

B

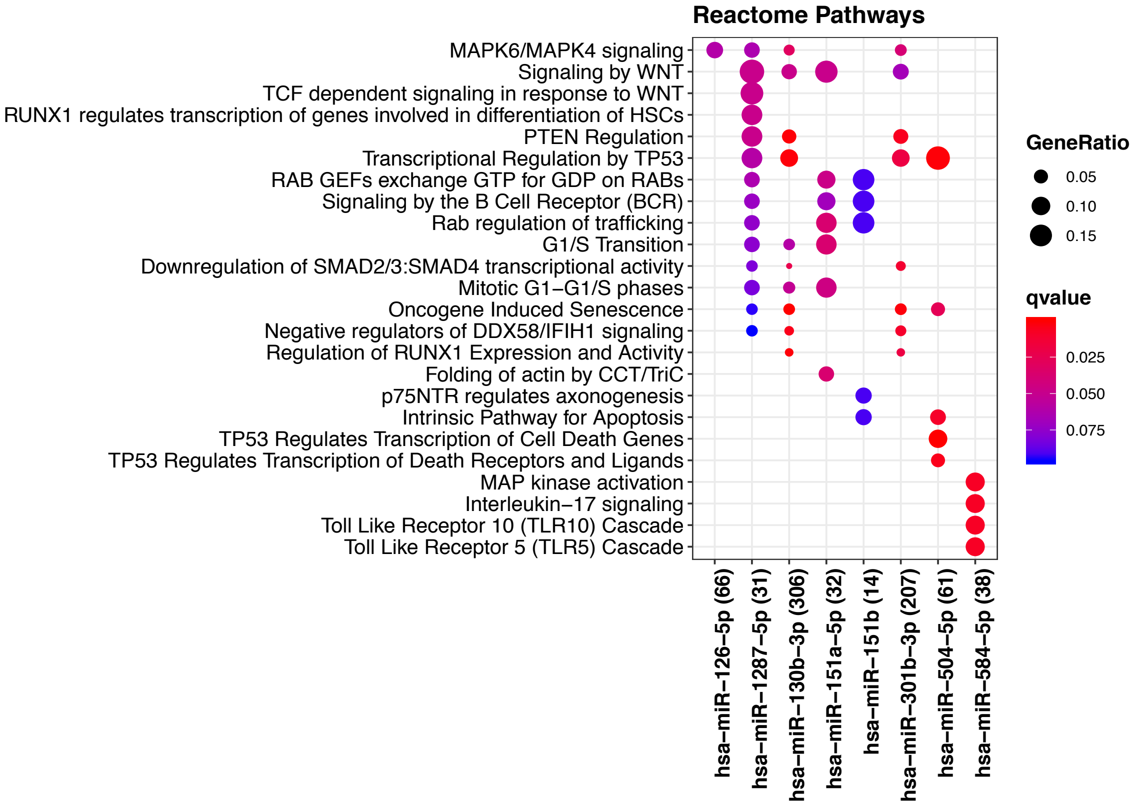

Figure S2. Functional enrichment analysis of genes targeted by downregulated miRNAs.

(**A-B**) The dotplot shows the top significantly enriched categories of GO biological processes of the differentially downregulated miRNAs target genes. The total number of target genes found in miRTarBase database for each miRNA is reported on x axis labels. Size dots is correlated with the ratio between the total number of target genes and the number of genes that belongs to a GO (**A**) or Reactome (**B**) category. Dots are colored according the Benjamini-Hochberg false discovery rate adjusted p-values (q value) from blue (higher q value) to red (lower q value).

Supplementary Figure S3

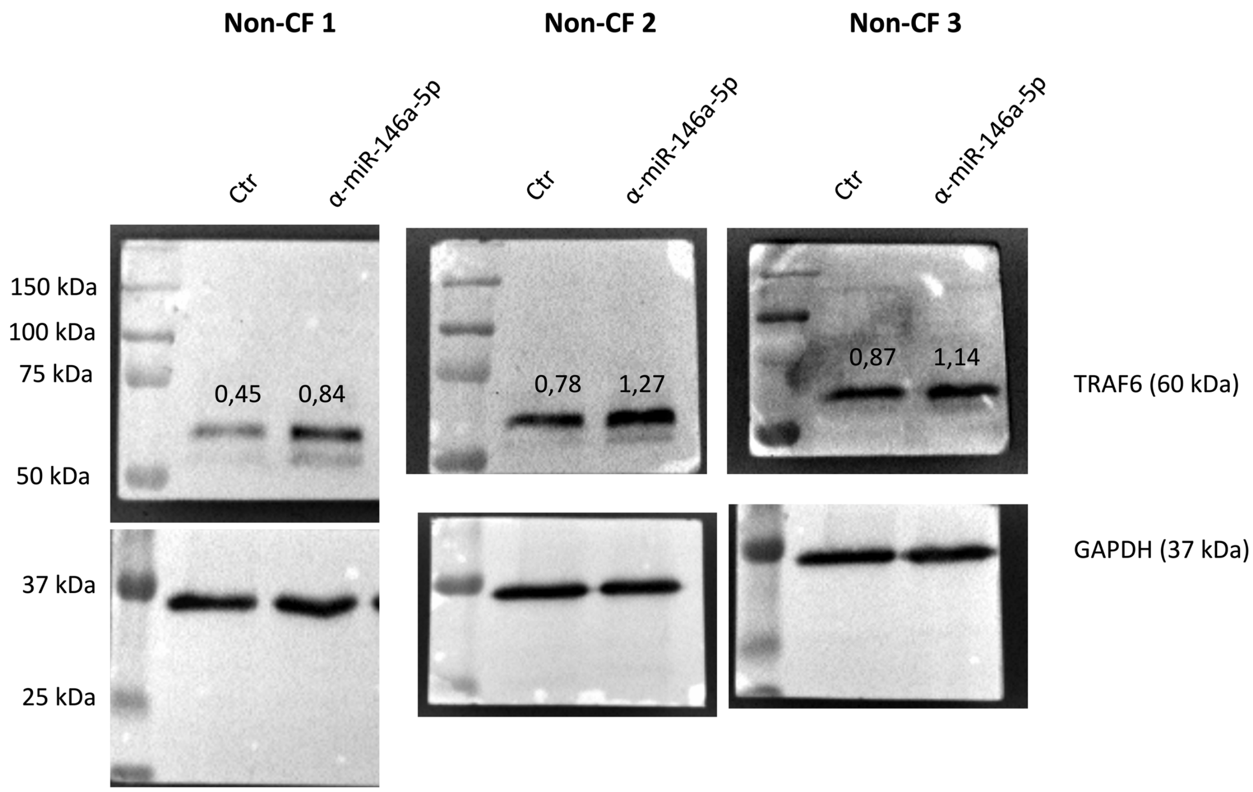

Figure S3. Uncropped western blotting images and quantification data of TRAF6 used for Figure 4b.
